# DORIE: Dataset of Road Infrastructure Elements—A Benchmark of YOLO Architectures for Real-Time Patrol Vehicle Monitoring

**DOI:** 10.3390/s25216653

**Published:** 2025-10-31

**Authors:** Iason Katsamenis, Nikolaos Bakalos, Andreas Lappas, Eftychios Protopapadakis, Carlos Martín-Portugués Montoliu, Anastasios Doulamis, Nikolaos Doulamis, Ioannis Rallis, Dimitris Kalogeras

**Affiliations:** 1 Institute of Communications and Computer Systems, 9 Ir. Politechniou, 15773 Athens, Greece; bakalosnik@mail.ntua.gr (N.B.); adrlappas@ntua.gr (A.L.); adoulam@cs.ntua.gr (A.D.); ndoulam@cs.ntua.gr (N.D.); irallis@central.ntua.gr (I.R.); d.kalogeras@noc.ntua.gr (D.K.); 2 Department of Applied Informatics, University of Macedonia, 156 Egnatia Street, 54636 Thessaloniki, Greece; eftprot@uom.edu.gr; 3 Acciona Construccion Technology Centre, 8 Valportillo II, 28108 Madrid, Spain; carlos.martinportugues.montoliu@acciona.com

**Keywords:** road infrastructure monitoring, traffic safety, deep learning, computer vision, YOLO, small object detection

## Abstract

Road infrastructure elements like guardrails, bollards, delineators, and traffic signs are critical for traffic safety but are significantly underrepresented in existing driving datasets, which primarily focus on vehicles and pedestrians. To address this crucial gap, we introduce DORIE (Dataset of Road Infrastructure Elements), a novel, high-resolution dataset specifically curated for real-time patrol vehicle monitoring along the A2 motorway in Spain. DORIE features 938 manually annotated images containing over 6800 object instances across ten safety-critical categories, including both static infrastructure and dynamic traffic participants. To establish a robust performance benchmark, we conducted an extensive evaluation of the YOLO family of detectors (versions 8, 11, and 12) across multiple scales and input resolutions. The results show that larger YOLO models and higher-resolution inputs yield up to 40% improvement in mean Average Precision (mAP) compared to smaller architectures, particularly for small and visually diverse classes such as traffic signs and bollards. The inference latency ranged between 5.7 and 245.2 ms per frame, illustrating the trade-off between detection accuracy and processing speed relevant to real-time operation. By releasing DORIE with detailed annotations and quantitative YOLO-based baselines, we provide a verifiable and reproducible resource to advance research in infrastructure monitoring and support the development of intelligent road safety and maintenance systems.

## 1. Introduction

Road infrastructure plays a crucial role in ensuring traffic safety, efficient mobility, and timely maintenance of transportation networks [1]. Continuous monitoring of roads is essential to detect damage, identify hazards, and support intelligent transportation systems [2]. Maintaining an up-to-date registry is central to infrastructure management. It supports safety (for example, verifying guardrail continuity and sign visibility), regulatory compliance (presence, legibility, and siting of required signs; correct delineation in work zones), lifecycle planning (linking condition and exposure to maintenance scheduling and budgeting), change detection and auditing after incidents or works, and the upkeep of digital twins and GIS layers used by decision-support systems [3]. Similar needs arise in other domains—railway furniture and signals, utility street furniture, street lighting, barriers and fences, and roadside IoT—so methods and datasets for vision-driven inventorying are broadly useful beyond highways [4,5].

Recent advances in computer vision and deep learning, combined with the availability of large annotated datasets, have enabled automatic analysis of road scenes for applications such as autonomous driving, traffic surveillance, and infrastructure maintenance [6,7,8]. While several benchmark datasets exist in the domain of autonomous driving and urban scene understanding, such as KITTI [9], Cityscapes [10], and Mapillary Vistas [11], they are typically designed for tasks like semantic segmentation or object detection of vehicles and pedestrians. However, they often underrepresent infrastructure-related elements such as guardrails, bollards, delineators, and specific traffic signs, which are of high importance for road safety assessment [12]. This fact, combined with the inherent challenges of detecting small, visually diverse, and sometimes occluded objects, makes existing benchmarks unsuitable for developing robust, real-time infrastructure monitoring systems [13].

To address these gaps, we introduce DORIE—the Dataset of Road Infrastructure Elements—a high-resolution, patrol-vehicle-centric object detection dataset focused on safety-critical, static roadside assets and broad traffic-sign super-categories relevant to inventorying workflows. DORIE is designed to match the operational viewpoint of patrol vehicles and emphasizes small and elongated objects common in asset registries. The dataset is publicly available at the following link: https://doi.org/10.5281/zenodo.17277466. The dataset specifically focuses on infrastructure-related objects and road safety elements. It is designed to facilitate the training and evaluation of object detection models targeting classes that are underrepresented in existing datasets. To provide initial benchmarks, we conduct extensive experiments with state-of-the-art object detection models from the You Only Look Once (YOLO) algorithm family (and in particular recent state-of-the-art YOLO versions 8, 11, and 12) under different input resolutions and model scales [14,15,16,17].

The rest of the paper is structured as follows. Section 2 discusses existing road monitoring datasets and object detection methods, with a focus on YOLO-based architectures, to contextualize our work. In Section 3, we describe the DORIE dataset, including its collection, annotation process, and key statistics. Section 4 presents the experimental evaluation, detailing the comparative models, metrics used, and the results of our performance analysis. Finally, Section 5 provides a discussion of our findings, and Section 6 concludes the paper with a summary of the contributions and directions for future research.

## 2. Related Work

Research on road scene understanding and monitoring has produced a wide range of datasets and detection methods, reflecting the growing interest in autonomous driving, intelligent transportation systems, and urban safety [18]. The present section reviews representative datasets that have shaped the field, as well as object detection approaches with an emphasis on YOLO-based architectures, in order to contextualize the contributions of our work.

### 2.1. Existing Road Monitoring Datasets

Over the past decade, the rapid advancement of intelligent transportation systems and autonomous vehicle technology [19] has spurred the creation of numerous large-scale datasets. These resources are fundamental to the development and evaluation of computer vision systems used in autonomous driving and urban scene analysis. The purpose of these datasets is to provide a standardized basis for training and testing algorithms, helping to advance the field by allowing researchers to benchmark their methods against one another. While some early datasets focused on specific aspects of the driving environment, such as pedestrian detection, the trend has shifted towards more comprehensive, multimodal datasets that capture a wide array of driving conditions and sensor data.

More specifically, some datasets, such as those from [20,21,22,23,24,25,26,27], concentrate on specific objects like pedestrians. In parallel, various datasets, including KITTI [9], nuScenes [28], and RobotCar [29], introduced tasks such as stereo, optical flow, and 3D object detection by incorporating data from multiple sources, like LiDAR point clouds. Other well-known datasets, including Cityscapes [10] and SYNTHIA [30], offer detailed 2D semantic segmentation for individual objects in video frames, offering large-scale pixel-level annotations for urban scenes, while Mapillary Vistas [11] offers highly diverse, user-uploaded data with detailed annotations, which addresses the issue of location diversity. Lastly, given the rise of autonomous driving, $D^{2}$-city [31] and ApolloScape [32] are large-scale driving video datasets, which deeply reflect the complexity and diversity of street views and real-world traffic scenarios.

Research into detecting and recognizing traffic signs has been a popular area of study [33]. The German Traffic Sign Benchmark Dataset (GTSBD) was one of the first resources created to test classification algorithms [34]. This was followed by the development of datasets for specific regions, such as those for Swedish [35], Belgian [36], and Russian signs [37], as well as the benchmark Tsinghua-Tencent 100K for China [38]. For the more general problem of detecting a traffic sign without identifying its specific class, more recently, researchers have exploited datasets, like BDD100K [39], which contains US-based signs and has expanded the diversity of driving conditions by including nighttime, weather variations, and different geographic locations.

Despite their impact, the aforementioned datasets often focus primarily on dynamic traffic participants, such as cars, trucks, bicycles, and pedestrians, with limited emphasis on static infrastructure elements. Objects such as bollards, delineators, or road guardrails are either absent, grouped into generic categories, or underrepresented, making them less suitable for infrastructure monitoring tasks.

### 2.2. Object Detection for Road Scene Understanding

Deep learning-based object detectors, particularly the convolutional neural network-based YOLO family, have gained significant popularity due to their efficiency and high accuracy in real-time applications [40]. Variants of YOLO have been widely adopted for traffic sign recognition, vehicle detection, and pedestrian monitoring. However, the detection of small and visually diverse infrastructure-related objects remains a challenging problem, as such objects often occupy only a few pixels in typical driving scene images.

On the one hand, there are lightweight approaches, focused on the YOLOv5 architecture, optimized for mobile devices [41]. Their approach was validated through experiments on the RDD-2020 road damage dataset. Given that a mobile device can be held or placed on the top of a trunk, such mechanisms can be beneficial. However, utilization over UAV or in scenarios where the scene is far from the capturing device is expected to lose multiple small-scale defects [42].

On the other hand, a multi-scale YOLO algorithm designed for object detection in complex road scenes, focusing on extremely small traffic objects often missed in dense environments, could be beneficial [43]. The specific implementation adds a fourth detection head for small objects and utilizes a new SPD-Conv CNN Module for better performance with low-resolution images. The dataset, collected from the ring highways of Xi’an in November 2021, comprises videos transformed into approximately 8000 images, capturing a diverse range of traffic objects.

Generally speaking, the YOLO family offers flexibility in road monitoring applications due to the architectural parameterization capabilities. The parameterization and custom-built layers allow for increased sensitivity to small-scale objects, or handling complex environments [44,45].

Beyond these representative approaches, a wide variety of object detection strategies for road scene understanding have been proposed in the literature, ranging from transformer-based detectors [46] to hybrid methods [47] integrating temporal or multimodal information [48]. While such directions serve important and diverse purposes, they extend beyond the scope of the present work, which focuses primarily on YOLO-based solutions.

### 2.3. Our Contribution

Inspired by the above research work, this paper addresses the significant gap in existing datasets by introducing DORIE (Dataset of Road Infrastructure Elements), a novel and highly specific dataset for road infrastructure monitoring. More specifically, the distinct innovation of this study lies in directly addressing the operational challenges of existing intelligent transportation systems data and methods. By creating the DORIE dataset and benchmarking state-of-the-art YOLO architectures, we move beyond the common focus on dynamic actors (like vehicles and pedestrians) to solve the critical, often-neglected challenge of static asset inventory. Our detailed analysis provides quantifiable metrics on the latency–accuracy trade-off for highly constrained small-object detection tasks, offering a reproducible framework for deploying real-time, patrol-vehicle-based digital twinning and safety assessment solutions. This shift in focus, coupled with our comparative analysis on input resolution, represents a core contribution toward making intelligent infrastructure monitoring technically feasible and economically viable.

To this end, unlike most popular datasets that focus heavily on dynamic elements like vehicles and pedestrians, DORIE is purposefully curated to emphasize safety-critical static infrastructure categories that are often underrepresented or absent in other collections, such as bollards, delineators, guardrails, and various traffic signs. To validate the usefulness of our dataset and establish a foundational benchmark for future research, we conduct a comprehensive evaluation of multiple YOLO architectures (versions 8, 11, and 12) across different scales and input resolutions. The experimental results offer a detailed analysis of the trade-offs between model size, inference speed, and detection accuracy, particularly for small and challenging objects.

The main contributions of this paper can be summarized as follows:

- We introduce DORIE, a new, high-resolution dataset of 938 manually annotated images with over 6800 object instances, collected from patrol vehicles on the A2 motorway in Spain.
- We provide a standardized benchmark by evaluating the performance of the YOLO family of detectors, which highlights the challenges of detecting small objects and underscores the necessity of higher resolution inputs for robust performance.
- By making DORIE publicly available, we provide the research community with a dedicated resource that directly supports the development of advanced algorithms for intelligent road safety and maintenance systems.

## 3. Dataset Description

As already mentioned in the previous sections, DORIE is designed to facilitate the development of computer vision algorithms for road infrastructure monitoring. The dataset, originating from Spanish roads, features a comprehensive array of scenes captured under real-world driving conditions. A particular focus is placed on infrastructure-related objects that are critical for road safety. It is noted that this dataset was curated and manually annotated as part of the H2020 HERON project, which sought to establish an integrated automated system for performing road maintenance and upgrading tasks [49,50]. The dataset is made publicly available to the research community at the following link: https://doi.org/10.5281/zenodo.17277466.

### 3.1. Data Collection Setup

**Figure 1:**
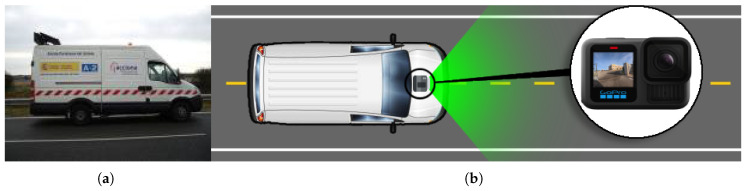
Experimental setup for acquiring RGB road images. (**a**) The utilized inspection vehicle during the data capturing process; (**b**) Schematic illustration of the optical sensor’s mounting location on the vehicle.

DORIE was collected using a GoPro HERO13 Black (GoPro, Inc., San Mateo, CA, USA) mounted on the hood of a patrol vehicle (see Figure 1). The camera was set to capture at 1 frame per second, recorded at a fixed forward-facing angle, and simulated the view of a typical dash camera. The images were captured at a resolution of 5568 × 4872 pixels, ensuring high-quality data. The acquisition platform ensured stable image capture, while GPS and timestamp metadata were also stored to enable potential extensions such as spatio-temporal analysis.

For complete reproducibility and to address the critical impact of imaging conditions on small objects (e.g., traffic signs, delineators, and bollards), the detailed sensor and lens parameters are provided. The imaging chain was configured using the highest photo resolution mode, providing the following specifications: the camera uses an electronic rolling shutter, which is common in high-resolution video sensors, and the fast exposure time of 1/4,129 s effectively minimizes the associated motion blur artifact, even at patrol speeds. The lens configuration corresponds to the wide Field of View (FOV), resulting in the 2.71 mm focal length. Furthermore, the standard processing pipeline ensures minimal interference: electronic image stabilization was off (as it typically reduces the FOV or resolution), and lens distortion correction was set to its standard digital correction setting. The use of the normal exposure program and average metering mode provides a consistent baseline for scene illumination, while the F-number of f/2.5 maintains a fixed, medium aperture. This detailed context fully supports the analysis of small and distant objects captured under typical daytime conditions.

**Figure 2:**
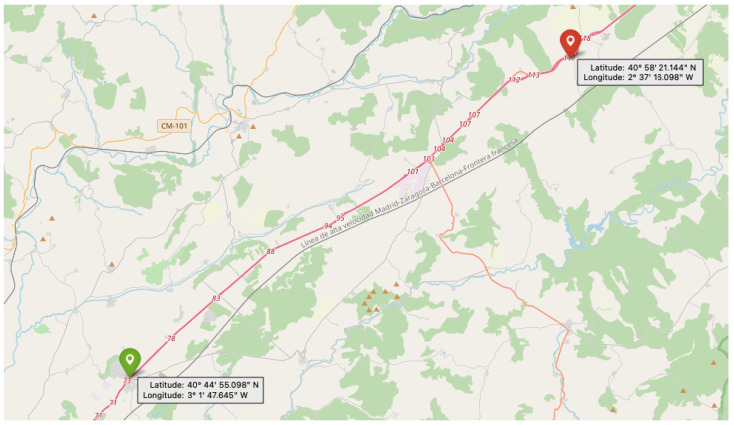
Map of the A2 motorway section in Spain where RGB data was acquired. The green pin marks the starting point of the patrol vehicle, and the red pin indicates the endpoint.

The data for this study were acquired from a 77.5 km stretch of the A2 motorway in Spain, which runs from Madrid to the boundary between the provinces of Guadalajara and Soria. The exact part of the road where the data acquisition took place is maintained by Acciona Construction S.A. and is shown in the map below (see Figure 2). The A2 is a critical transportation artery connecting Madrid and Barcelona, forming part of both the TEN-T (Trans-European Transport Network) and the CEF (Connecting Europe Facility) corridor, a key EU transport route that connects major cities and hubs across member states to ensure seamless and sustainable mobility. The selected section is a four-lane road (two in each direction) that experiences high traffic levels and a Continental–Mediterranean climate, with long, severe winters and hot, dry summers.

### 3.2. Classes and Annotations

The dataset covers a range of categories directly related to infrastructure monitoring and road safety that are critical for a comprehensive understanding of the road environment. The annotated classes include the following:

- Bollard (Bol): Roadside safety posts or poles.
- Delineator (Del): Reflective roadside markers used to guide traffic.
- Prohibitory sign (Pro): Standard traffic prohibition signs.
- Danger sign (Dan): Warning signs indicating hazards ahead.
- Mandatory sign (Man): Signs prescribing specific driving actions.
- Other sign (Oth): Additional traffic signs not falling into the above categories.
- Car (Car): Passenger vehicles visible on the road.
- Truck (Trk): Larger vehicles such as lorries and freight trucks.
- Guardrail (Grd): Roadside safety barriers.
- Road (Rd): The drivable road surface.

**Figure 3:**
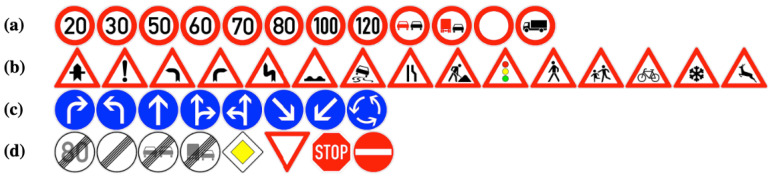
The four different categories of traffic signs. (**a**) Prohibitory; (**b**) Danger; (**c**) Mandatory; and (**d**) Other signs.

It should be emphasized that the traffic signs were systematically grouped following the taxonomy proposed by Houben et al. [51], which provides a well-established framework for structuring European traffic sign datasets. This grouping ensures consistency with prior benchmarks and facilitates comparability with existing detection studies. As illustrated in Figure 3, the first category, prohibitory, comprises circular signs with a white background and red border; the second, danger, includes triangular signs also featuring a white background and red border; the third, mandatory, consists of circular signs with a blue background; and the final category, other, encompasses all remaining traffic signs that do not fall into the aforementioned groups.

Lastly, it is noted that all images were manually annotated with bounding boxes using a dedicated annotation tool. A quality control process was applied to ensure label consistency and correctness. In particular, the points of interest were initially annotated by a junior computer vision engineer with 3 years of experience. This initial dataset was then refined by a team of three engineers with 5 to 15 years of experience to improve accuracy and consistency. Finally, a two-stage quality assurance process was implemented, with all annotations undergoing further refinement and verification by two senior engineers with more than 25 years of experience in the specific or a related field. Difficult cases, such as partially occluded or distant objects, were reviewed by multiple annotators to minimize errors. In this hierarchical review workflow, higher-level annotators directly corrected or replaced annotations identified as inaccurate, thereby ensuring that only the most reliable annotations were retained. As previous annotations were overwritten rather than preserved, explicit inter-annotator agreement metrics could not be computed. Nonetheless, this tiered validation procedure minimized label noise and ensured a consistent, high-quality final dataset through cumulative expert refinement.

### 3.3. Dataset Statistics

The dataset contains a total of 938 annotated images, with approximately 6852 object instances. As already mentioned, the images are provided at a native resolution of 5568 × 4872 pixels. To facilitate model development and evaluation, the dataset is split into training, validation, and test subsets. More specifically, an 80% subset was allocated for combined training and validation purposes, while the remaining 20% was reserved for the test set. Within the training and validation subset, a further 80/20 split was applied to create the final training and validation data partitions, respectively. The total per-class distribution, as well as for each of the subsets, is summarized in Table 1.

To ensure spatial coverage of the motorway, which is illustrated in Figure 2, the dataset split was designed so that all route sections are represented across the training, validation, and test subsets. This frame-based sampling strategy prioritizes spatial generalization, enabling the models to encounter the full variety of infrastructure types, backgrounds, and lighting conditions present along the route. Although sequential acquisition can lead to limited visual overlap between subsets, this approach was intentionally chosen to maintain a balanced distribution of object categories. Future extensions of DORIE will consider contiguous route or time-block splits to further quantify generalization performance across geographically disjoint segments.

As can be seen in Table 1, classes like delineator and guardrail are the most prevalent, with 1896 and 1635 instances, respectively. In contrast, certain categories are much less common, with the danger signs having only 105 instances and the mandatory signs having 131 instances. This class imbalance presents a significant challenge for object detection methods, as they must learn to accurately identify both frequently and sparsely represented points of interest.

**Figure 4:**
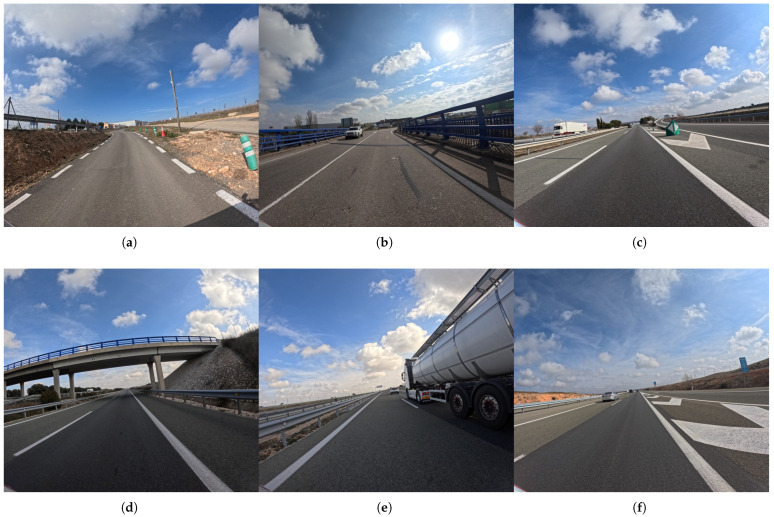
Representative samples from the DORIE dataset. (**a**–**f**) Indicative images showcasing the diversity of road environments, weather conditions, and scenes captured within the dataset.

In Figure 4, representative examples of annotated images are shown, illustrating the diversity of classes, viewpoints, and scene complexity. As one can observe, the collection includes variability in lighting and weather, from bright sunny backgrounds to scenes with high sun glare that can partially obscure objects. Furthermore, the images present common challenges such as occlusion, where objects like bollards or traffic signs are partially blocked by other vehicles or environmental elements. The dynamic nature of the traffic is also captured, with varying speeds of the patrol vehicle affecting the size and clarity of objects. Hence, these scene complexities make our dataset a valuable resource for training and testing algorithms for robust road infrastructure monitoring.

**Figure 5:**
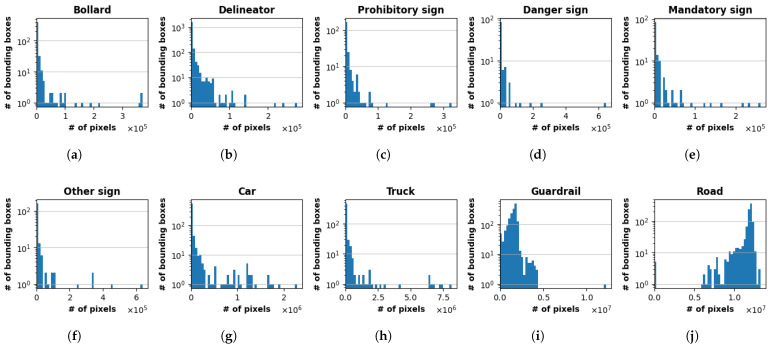
Distribution of bounding box sizes for each of the 10 classes. (**a**) Bollard; (**b**) Delineator; (**c**) Prohibitory sign; (**d**) Danger sign; (**e**) Mandatory sign; (**f**) Other sign; (**g**) Car; (**h**) Truck; (**i**) Guardrail; and (**j**) Road. The *y*-axis is displayed on a logarithmic scale (base 10) to clearly show the frequency of smaller boxes.

The histograms presented in Figure 5 illustrate the distribution of bounding box sizes, measured in pixels, for each of the 10 object classes in our dataset. A strong common trend is observed across most classes, which exhibit a long-tailed distribution with a high concentration of small bounding boxes. This pattern is particularly evident for classes such as bollard, delineator, and sign-related categories, where the majority of instances are very small and likely represent distant objects. The vehicle classes (i.e., car and truck) follow a similar trend, but with a broader range of sizes and a more scattered distribution of larger boxes, reflecting their varying scale in the images, as seen for instance in Figure 4e compared to (f). On the other hand, it is underlined that the road class presents a distinct, almost bimodal distribution, showing a small cluster of low-pixel-count boxes and a dominant concentration of very large boxes exceeding one million pixels. This suggests a mix of small road segments and very large instances that span the full width of the images. Similarly, the guardrail class also displays a unique pattern, characterized by a long tail and a significant single instance outlier representing a very large bounding box.

## 4. Experimental Evaluation

### 4.1. Comparative Models—Experimental Setup

The selection of an appropriate object detection model for road infrastructure applications is critically dependent on achieving an optimal balance between detection accuracy and inference speed. This study focuses on comparing three recent generations of the YOLO (You Only Look Once) family [14], and in particular YOLOv8, YOLOv11, and YOLOv12, across their various scales (nano, small, medium, and large) to determine the most effective architecture for detecting the road elements that were presented in Section 3.2.

YOLOv8, released by Ultralytics, laid a powerful foundation for the subsequent versions [15]. It represents an optimized, anchor-free network design, introducing advanced backbone and neck architectures alongside a split detection head [52]. This architectural shift marks a significant step toward an improved accuracy–speed trade-off compared to its predecessors [53]. The YOLOv8 family serves as a critical baseline in various computer vision experiments, with its efficient design maximizing inference speed while maintaining robust detection performance [54,55,56].

Building upon YOLOv8’s success, YOLOv11 introduced architectural refinements aimed specifically at efficiency and performance scaling [16]. Key to YOLOv11’s improvements is an enhanced feature extraction framework that leads to greater mean Average Precision (mAP) while simultaneously reducing the model’s complexity [57]. For instance, the YOLOv11 medium variant achieves superior accuracy on the COCO dataset compared to YOLOv8 medium, yet utilizes approximately 22% fewer parameters [58]. These optimizations underscore a continued focus on efficient deployment, ensuring the model can run reliably on diverse platforms, including edge devices commonly found in real-world road monitoring systems. The incremental gains in feature processing and parameter efficiency are crucial for deploying models in real-time embedded environments where computational resources are constrained [59].

YOLOv12 represents the most significant architectural departure in this comparison, shifting toward an attention-centric design while preserving the real-time inference capability synonymous with the YOLO lineage [17]. This iteration replaces much of the traditional CNN-based structure with novel attention mechanisms [60]. The central innovation is the Area Attention Mechanism, a self-attention approach that processes large receptive fields by dividing feature maps into distinct, equally sized regions, which drastically reduces computational overhead compared to standard self-attention [61]. This is coupled with Residual Efficient Layer Aggregation Networks (R-ELAN), which address optimization challenges inherent in larger attention models through block-level residual connections [62].

The comparative analysis across the nano, small, medium, and large versions of each model is designed to provide a comprehensive view of how architectural advancements scale with model size. By examining the performance of these model variants on a custom road infrastructure dataset, this experimental framework directly quantifies the impact of YOLOv8’s anchor-free head, YOLOv11’s parameter-efficient feature enhancement, and YOLOv12’s attention-based structure on the accuracy, speed, and overall computational cost associated with detecting complex and varied road objects. The number of parameters and Floating-point Operations (FLOPs) of the models, detailed in Table 2, serve as the foundation for quantifying the inherent trade-off between model size and the resulting computational intensity of each architecture variant.

Lastly, it is highlighted that the aforementioned detectors were trained and evaluated using an NVIDIA Tesla T4 GPU with 12 GB of memory. For consistency, all networks were trained for 100 epochs using a batch size of 8. Crucially, the training and evaluation procedures were performed at two distinct input image resolutions, 576 × 640 pixels and 1120 × 1280 pixels, specifically to investigate and quantify the impact of input resolution on detection accuracy and computational overhead across the different architectural scales.

### 4.2. Performance Metrics

Performance metrics for object detection tasks are built upon four fundamental concepts: true positives (TP), which are correct detections; false positives (FP), which are incorrect detections; false negatives (FN), which are missed detections; and true negatives (TN), which are correctly identified backgrounds. A key factor in determining a TP is the Intersection over Union (IoU), which must exceed a specified threshold. These concepts are used to derive other metrics: Precision measures the accuracy of positive predictions by calculating the ratio of TPs to the total number of detections. Recall assesses the model’s ability to find all relevant instances by calculating the ratio of TPs to the total number of actual instances. The F1-score is a single metric that provides a harmonic mean of precision and recall, balancing the trade-off between minimizing false alarms and missed detections to offer a comprehensive measure of overall model performance. The aforementioned metrics are calculated as follows:

(1) $$\begin{array}{c} Prec=\frac{TP}{TP+FP},\;Rec=\frac{TP}{TP+FN},\;F1=\frac{2\cdot Prec\cdot Rec}{Prec+Rec}=\frac{TP}{TP+\frac{1}{2}\left(FP+FN\right)} \end{array}$$

In parallel, the Average Precision (AP) is a key metric that summarizes a model’s performance for a single class by calculating the area under its precision–recall curve, represented as p(r). This curve plots a model’s precision against its recall at different confidence thresholds. A higher AP value signifies a model that is both accurate and reliable for a specific object class. To obtain a single value for the model’s overall performance across all object classes, the Mean Average Precision (mAP) is used. This metric is simply the average of the AP scores for each individual class, providing a comprehensive assessment of the model’s ability to detect and localize objects across the entire dataset. Variants such as mAP50 (IoU threshold of 0.5) emphasize lenient localization, while mAP50:95 averages across thresholds for a stricter evaluation. The formulas for AP and mAP, where N is the total number of classes, are given by the following:

(2) $$\begin{array}{c} AP=\int_{0}^{1}p\left(r\right)\;dr,\;mAP=\frac{1}{N}\sum_{k=1}^{N}AP_{k} \end{array}$$

### 4.3. Experimental Results

#### 4.3.1. mAP Analysis

This section presents a detailed analysis of the experimental results obtained from evaluating various YOLO models, specifically versions 8, 11, and 12, each trained with nano, small, medium, and large architectures, on our dataset. The performance of each model was measured using the metrics presented in Section 4.2. The following analysis is based on the mAP50 scores, and, more specifically, Table 3 presents the results for all models evaluated with an input size of 576 × 640.

Overall, the results indicate a clear positive correlation between model size and detection performance. In particular, the YOLOv8l model consistently demonstrated superior performance, achieving the highest overall mAP50 score of 60.7%. This trend suggests that for the given dataset, the increased parameter count and complexity of larger models, such as the medium and large variants, are crucial for improving overall detection accuracy. This is particularly evident when comparing the nano YOLO models, which exhibit the lowest overall scores, with their deeper counterparts, where performance gains of over 11% are observed for all the YOLO versions.

It is also noted that a class-by-class analysis reveals significant variations in detection performance. More specifically, classes such as guardrail and road consistently achieved exceptionally high mAP50 scores across all models, often exceeding 97%. This is likely due to the size, distinct shape, and consistent appearance and colors of these objects, which makes them relatively easy to identify and localize. Similarly, truck detection was highly accurate, with the YOLOv8l model reaching a remarkable mAP50 of 93.6%. These findings highlight the models’ proficiency in detecting large, well-defined objects with predictable visual characteristics.

Conversely, the performance for smaller, less common, or visually challenging classes varied considerably. Danger sign proved to be the most difficult class to detect, with all models yielding very low mAP50 scores, ranging from 4.49% for YOLOv11n to a peak of 31.0% for YOLOv8l. This difficulty could be attributed to the often-small size of these signs in the images, their varied visual appearance, and potential occlusions. Similarly, classes like bollard, delineator, and other sign showed lower scores in the nano models but saw substantial performance improvements with the small, medium, and large architectures, underscoring the benefit of greater model capacity for these challenging identifications.

To this end, a key factor contributing to the low mAP50 scores for certain classes, particularly those of signs, is the small size of these objects within the 576×640 input resolution. At this resolution, small objects like signs and bollards occupy a very limited number of pixels, making it difficult for the models to extract the necessary features for accurate classification and localization. The limited pixel information results in a significant reduction in detection accuracy, which is reflected in the low scores for danger sign and the relatively modest scores for bollard and other sign across all model architectures.

To investigate the impact of input resolution on model performance, a second set of experiments was conducted using an input size of 1120×1280. The results regarding the mAP50 metric are presented in Table 4.

As can be seen in the aforementioned Table, the results from the higher input resolution detectors continue to support the trend that larger architectures achieve better performance. The YOLOv8l model again emerged as the top performer, achieving an overall mAP50 of 86.2%. The consistent outperformance of larger networks, particularly for the more challenging classes, highlights the benefit of increased parameter capacity when dealing with more detailed visual information. This is especially apparent in the medium and large architectures, which leverage the increased pixel density to better recognize and classify the various points of interest.

In parallel, it is emphasized that the most significant performance gains at the larger 1120×1280 resolution were observed in the classes that struggled with the lower resolution. Notably, the danger sign class, which previously had the lowest scores (see Table 3), saw its mAP50 for YOLOv8l jump from 31.0% to a remarkable 92.7%. Similarly, bollard and delineator showed substantial improvements across all model sizes. Thus, the enhanced resolution provides the models with a greater number of pixels to analyze for these small objects, allowing for a more accurate feature extraction and, consequently, better detection. This validates the hypothesis that small object size was a primary limitation at the 576×640 input resolution.

The improvement in resolution had a considerable impact on the overall performance of all YOLO detectors. For instance, the YOLOv8l model showed a total mAP50 improvement of approximately 42%, increasing from 60.7% to 86.2%. The YOLOv11m model also exhibited a significant gain of about 45.3%, jumping from an overall score of 58.3% to 84.7%. It is noted that, while the benefits were less pronounced for already well-performing classes like guardrail and road, the higher resolution provided a significant boost to the overall utility and accuracy of the algorithms on DORIE, particularly for the challenging classes.

Building on the analysis of the mAP50 scores, we now turn our attention to the more rigorous mAP50:95 metric, which, as defined in Section 4.2, provides a more comprehensive evaluation of a model’s performance by averaging the Average Precision across a range of stricter Intersection over Union (IoU) thresholds. This metric particularly penalizes imprecise bounding box localization. The results for the YOLO models with a 576 × 640 input size are presented in Table 5.

The aforementioned results show a significant decrease in scores across all models and classes compared to the mAP50 metric. This is expected, as mAP50:95 averages performance over a much stricter range of IoU thresholds, from 0.5 to 0.95. Achieving a high mAP50:95 score requires not only accurate recognition and detection but also highly precise bounding box localization, which is a much more challenging task. The overall mAP50:95 scores for all models with a 576 × 640 input size range from 32.1% to 40.0%, indicating that the majority of models struggle to produce a highly accurate detection and a precise bounding box simultaneously.

Despite the overall performance drop, the trend of larger models outperforming smaller ones remains consistent. The YOLOv8m model leads the pack with an overall mAP50:95 of 40.0%, closely followed by the YOLOv8l model at 39.6%. The performance gains from increasing model size are evident across all YOLO versions, reinforcing the notion that greater model complexity is beneficial for more demanding tasks, such as precise localization. The performance gap between the nano and medium/large models is more pronounced under this metric, highlighting the struggle of lightweight models with the strict IoU requirements.

A class-by-class analysis further illustrates the challenges of the mAP50:95 metric. While classes like guardrail and road still maintain the highest scores, their performance remains nearly perfect, similarly to their results under the more lenient mAP50 metric. This indicates that for these large, distinct objects, the models are highly proficient at both accurate detection and precise bounding box localization. On the other hand, the truck and car classes experienced a significant drop, though they still remain among the better-performing categories, with the top scores for truck reaching just over 60%.

The most notable impact of the mAP50:95 metric is seen in the classes that were already difficult to detect at a lower resolution. Indicatively, the danger sign class, which had a peak mAP50 score of 31.0% (see Table 3), now registers a peak mAP50:95 score of just 14.3% for the YOLOv8l model. This dramatic reduction underscores the extreme difficulty of precisely localizing these small, inconsistent objects. In parallel, classes like bollard, delineator, and other sign also show a similar pattern, with their best scores falling to around 20% and below, revealing that models that could successfully detect these objects at mAP50 struggle to achieve the pixel-perfect bounding boxes required by mAP50:95. This strongly suggests that for small objects, the models’ ability to localize precisely is a major performance bottleneck.

In summary, the mAP50:95 results reinforce the findings from the mAP50 evaluation but with a greater emphasis on the challenges of precise localization. The consistent hierarchy of performance from larger to smaller models is maintained, with the YOLOv8m model demonstrating the best overall performance, closely followed by YOLOv8l. However, the significantly lower scores across all classes highlight the difficulty of the task, particularly for small objects, where both detection and localization are hindered by the limited pixel information.

The analysis of the 576 × 640 results highlighted the challenge of precisely localizing small objects, which was especially apparent under the stricter mAP50:95 metric. To investigate whether a higher input resolution could mitigate this issue, again, a second evaluation was performed using models with an input size of 1120 × 1280. Table 6 presents the mAP50:95 scores for this higher-resolution experiment.

The results from the higher resolution models continue to support the trend that larger models achieve better performance. The YOLOv8l and YOLOv11m networks emerged as the top performers, both achieving an overall mAP50:95 of 50.3%. This highlights that for this dataset, an increased input resolution significantly enhances the performance of more complex models. The consistent outperformance of medium and large variants over their smaller counterparts emphasizes the benefit of greater parameter capacity when precise localization is required.

A class-by-class analysis for the 1120 × 1280 resolution reveals that the most substantial performance gains were observed in the classes that struggled with the lower resolution. Notably, the danger sign class, which had the lowest scores previously, saw its peak mAP50:95 for YOLOv8l jump from 14.3% to a remarkable 55.2%, a gain of over 280%. Similarly, classes like bollard, delineator, and mandatory sign, which faced difficulties with precise localization, showed substantial improvements across all model sizes. This validates the hypothesis that small object size was a primary limitation at the 576 × 640 input resolution and that a higher resolution provides the models with the necessary pixel information for accurate feature extraction and localization.

The overall improvement in resolution had a considerable impact on the overall performance of all models. For instance, the YOLOv8l model showed a total mAP50:95 improvement of approximately 27%, increasing from 39.6% to 50.3%. The YOLOv11m model also exhibited a significant gain of about 30.6%, jumping from an overall score of 38.5% to 50.3%. While the benefits were less pronounced for already well-performing classes like guardrail and road, the higher resolution provided a significant boost to the overall utility and accuracy of the models on this dataset, particularly for the challenging classes.

In conclusion, the analysis of both mAP50 and mAP50:95 metrics confirms that model size and input resolution are critical factors in achieving high performance on object detection tasks. The consistent performance hierarchy from larger to smaller models is maintained across both metrics and resolutions. More importantly, the results show that while mAP50 is a useful indicator of general detection, the more stringent mAP50:95 metric highlights the importance of precise localization, a challenge that is significantly mitigated by increasing the input resolution. The remarkable improvement in scores for previously challenging objects like the various traffic signs underscores the necessity of providing sufficient visual information to the models for accurate and reliable performance.

#### 4.3.2. Precision and Recall Analysis

The analysis of aggregated mAP scores of the previous section provided a high-level overview of models’ performance, but a deeper understanding of their strengths and weaknesses requires a class-by-class examination of Precision and Recall. These metrics reveal the trade-offs models make between correctly identifying positive instances (recall) and minimizing false alarms (precision). The following tables (i.e., Table 7 and Table 8) present the F1-score, which is the harmonic mean of Precision and Recall, for both the lower 576 × 640 and higher 1120 × 1280 input resolutions, allowing for a thorough comparative analysis of each class.

A general overview of the results from the aforementioned tables indicates that models with an input resolution of 1120 × 1280 consistently achieve significantly higher Precision and Recall and thereby F1-scores across all classes compared to the 576 × 640 resolution. This is particularly noticeable in the average scores for all classes, where the higher-resolution models demonstrate a more balanced and effective performance. Additionally, the Precision and Recall scores, while often moving in tandem, sometimes show trade-offs for individual classes and models, a phenomenon that is explored in more detail in the class-specific analysis below.

**Figure 6:**
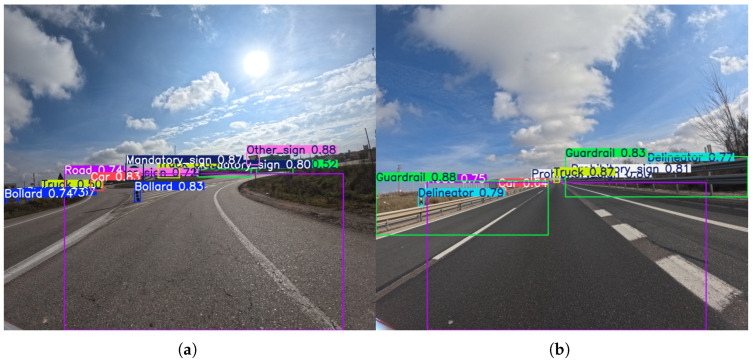
Representative outputs of the YOLOv8l model with an input size of 1120 × 1280, which demonstrates the best overall performance in detecting various road points of interest. An example (**a**) of object detection on a curved road section showing various points of interest, and (**b**) showing detection performance on a straight highway segment.

The data for both resolutions highlight a clear link between model size and overall performance. At the lower 576 × 640 resolution, the large and medium models tend to show a better balance between Precision and Recall than their nano and small counterparts. For instance, the YOLOv11l model achieves the highest overall Precision (87.0%) at this resolution, while the YOLOv8m model has the highest overall Recall (57.1%). The nano models, in contrast, consistently have lower scores, indicating a general struggle to achieve both high precision and high recall simultaneously. This pattern is even more pronounced at the higher 1120 × 1280 resolution (see Figure 6), where the top-performing models, such as YOLOv12s, YOLOv11m, and YOLOv8l, achieve a much better balance, with Precision and Recall scores both well above 75%.

Starting the comparative analysis of each class with the bollard class, at the 576 × 640 resolution, there is a clear trade-off between Precision and Recall. The nano and small models, such as YOLOv8n (95.6% Precision) and YOLOv8s (88.2% Precision), achieve very high precision scores, indicating that they are highly accurate when they do make a detection, with very few false positives. However, their Recall scores are very low (18.3% and 24.1%, respectively), meaning that they fail to detect a large majority of the actual bollards. In contrast, the YOLOv11m model, which has the highest Recall at this resolution (37.6%), does so with a lower but still respectable Precision of 77.7%. This pattern underscores the difficulty of accurately detecting this small, visually consistent object.

**Figure 7:**
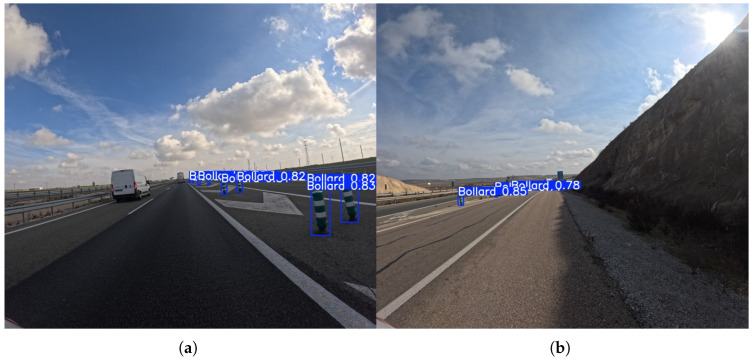
Representative outputs of the YOLOv11l model with an input size of 1120 × 1280, which demonstrates the best overall performance in the bollard detection task. An example (**a**) demonstrating successful detection of multiple bollards on a high-speed road, and (**b**) showing detection of bollards in a challenging low-light/high-contrast roadside environment.

As can be seen in Figure 7, increasing the input resolution to 1120 × 1280 dramatically improves performance for the bollard class across all models. Recall scores more than doubled for most architectures, with the highest Recall now reaching 74.2% (YOLOv11l). Precision scores also saw a general increase, and the volatility seen at the lower resolution was significantly reduced. This indicates that the models’ ability to both correctly identify bollards and find a higher proportion of them is directly proportional to the amount of pixel information available. The most substantial improvements were seen in the medium and large models, which capitalized on the higher resolution to achieve a better balance of Precision and Recall, with top scores in the high 80s for Precision and high 60s for Recall.

Subsequently, the delineator class at the 576 × 640 resolution shows a similar trade-off to bollards. Some models, such as YOLOv11l (95.2% Precision), are highly precise but less effective at finding all instances (26.0% Recall). The nano and small models consistently show low Recall scores, hovering around 20%, indicating that a significant number of delineators are missed. The best Recall score is only 30.7% (YOLOv8m), showing the general difficulty of this class for detection. This is likely due to their small size and vertical, often thin, structure, which can be easily missed or mistaken for noise.

**Figure 8:**
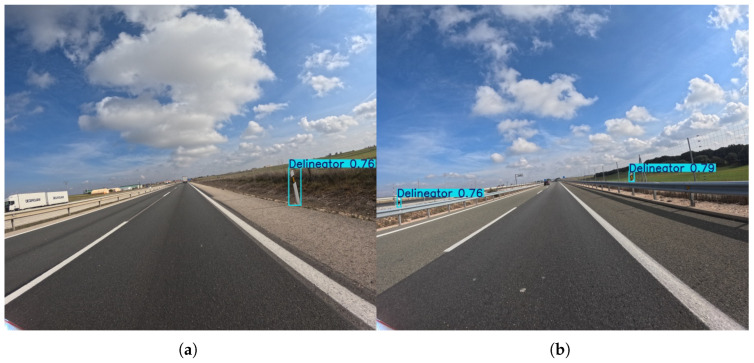
Representative outputs of the YOLOv11l model with an input size of 1120 × 1280, which demonstrates the best overall performance in the delineator detection task. An example (**a**) demonstrating successful detection of a single delineator in the near-field of the camera, and (**b**) showing detection performance on multiple delineators along a guardrail.

Again, as one can observe in Figure 8, when the resolution is increased to 1120 × 1280, the performance for delineator detection improves significantly. The highest Precision score climbs to a remarkable 93.9% (YOLOv12m), and the top Recall score nearly doubles to 56.9% (YOLOv8l). This indicates that the additional pixel information allows the models to not only locate delineators more effectively but also to classify them with a high degree of certainty, thereby reducing false positives. While the performance is not at the level of larger, more distinct objects, the gains clearly validate the importance of input resolution for accurately detecting small and visually challenging objects.

In parallel, at the 576 × 640 resolution, the prohibitory sign class presents a challenge for the models. Precision scores are generally in the range of 60–85%, with YOLOv11s achieving the highest precision at 84.9%. The Recall scores, however, are considerably lower, with the best score at 51.2% (YOLOv11m), indicating that a large portion of these signs are not being detected. This is a common pattern for small objects, where models opt for higher confidence to avoid false positives at the cost of missing true instances.

**Figure 9:**
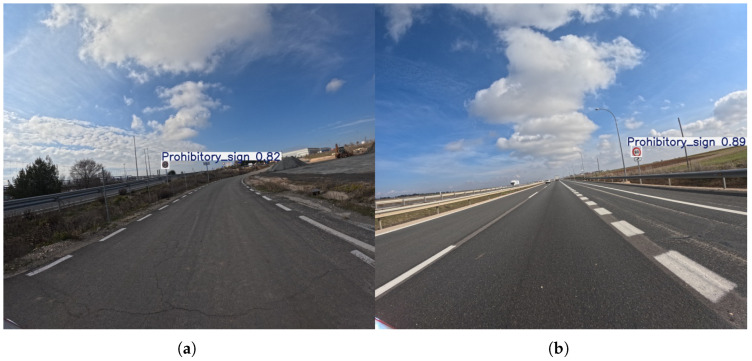
Representative outputs of the YOLOv8l model with an input size of 1120 × 1280, which demonstrates the best performance in the prohibitory sign detection task. An example (**a**) showing detection of a sign on an access road, and (**b**) showing detection of a speed limit sign on a highway.

With the resolution increase to 1120 × 1280, both Precision and Recall for prohibitory signs show marked improvements (see Figure 9). The best Precision score soars to 93.4% (YOLOv8m), and the peak Recall reaches 77.0% (YOLOv8l). This indicates that the higher resolution provides the models with the necessary detail to not only identify these signs more accurately but also to find a greater percentage of them. The improvement for this class is particularly significant, as the models are now much more capable of both classifying and locating these signs, reducing the trade-off that was prevalent at the lower resolution.

The danger sign class is the most difficult to detect at the 576 × 640 resolution, as evidenced by its extremely low mAP scores and analyzed in the previous paragraphs. This is directly reflected in its Precision and Recall scores. The models exhibit highly volatile Precision scores, with some dropping to as low as 6.3% (YOLOv12m), meaning that a very high percentage of its positive detections are incorrect. Recall is also abysmal, with YOLOv8m achieving a peak of just 30.4%, while other models miss almost every instance. This highlights the severe limitations of models when faced with very small, inconsistent, and often occluded objects.

**Figure 10:**
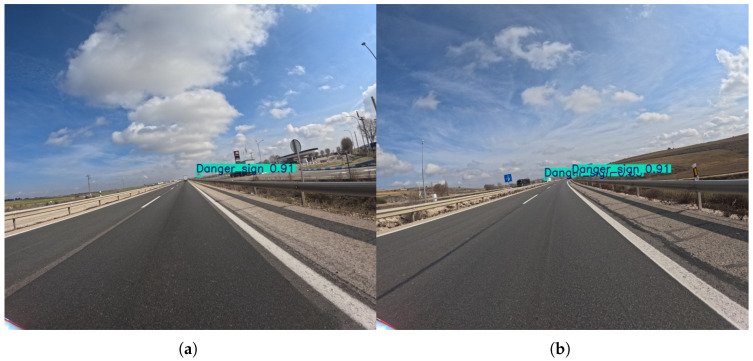
Representative outputs of the YOLOv8l model with an input size of 1120 × 1280, which demonstrates the best overall performance in the danger sign detection task. An example (**a**) demonstrating successful detection of a Danger sign positioned close to the roadside guardrail, and (**b**) showing detection of multiple Danger signs with a different background.

The resolution increase to 1120 × 1280 provides the most dramatic improvement for the danger sign class (see Figure 10). The Precision scores become much more stable and accurate, with several models reaching over 80%. Similarly, the Recall scores skyrocket across the board, with YOLOv8l achieving a remarkable 87.5%. The jump in performance is astounding, as a class that was nearly undetectable at the lower resolution is now identified with a high degree of confidence and effectiveness. This underscores that for extremely small and challenging objects, the input resolution is arguably the single most important factor for achieving robust performance.

It is noted, however, that at the 576 × 640 resolution, the mandatory sign class shows moderate performance. Recall scores are relatively consistent, with most models achieving scores in the mid-40s, and YOLOv11m reaching a high of 51.9%. Precision scores are more varied, with YOLOv8l achieving the highest score at 81.7%. The overall trend suggests that while models are somewhat successful at detecting these signs, there is still room for improvement in both finding all instances and avoiding false positives.

**Figure 11:**
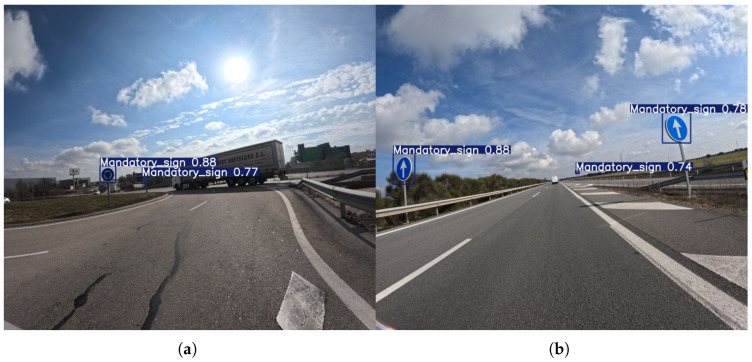
Representative outputs of the YOLOv8m model with an input size of 1120 × 1280, which demonstrates the best overall performance in the mandatory sign detection task. An example (**a**) demonstrating successful detection of Mandatory signs in a complex scenario involving sun glare and occluding vehicles (truck), and (**b**) showing detection performance on several Mandatory signs positioned at different distances on an open highway.

As presented in Figure 11, with the higher 1120 × 1280 resolution, the performance for mandatory signs improves notably. Both Precision and Recall scores increase significantly, with the YOLOv12s model achieving a peak Precision of 95.5%, and YOLOv11m achieving a peak Recall of 88.9%. This indicates that the additional pixel information allows the models to resolve the features of these signs more clearly, leading to a much more accurate and robust detection system. The increased resolution effectively mitigates the performance limitations seen at the lower resolution, making the models highly effective for this class.

The other sign class at a lower resolution is a challenging category due to its varied appearance and often small size. The Precision scores show high volatility, with a high of 97.0% for YOLOv8s and a low of 25.8% for YOLOv12m, indicating that some models are highly precise but miss many signs, while others make many false detections. Recall scores are uniformly low, with the best score at only 33.1% (YOLOv8m). This illustrates the models’ difficulty in both identifying and locating this diverse class of objects.

**Figure 12:**
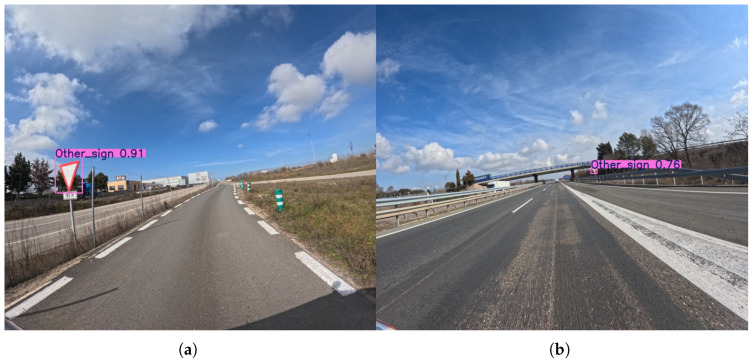
Representative outputs of the YOLOv8l model with an input size of 1120 × 1280, which demonstrates the best overall performance in the other sign detection task. An example (**a**) demonstrating successful detection of a sign on a low-speed access road adjacent to an industrial area, and (**b**) showing detection performance on a sign positioned on the side of the highway.

The 1120 × 1280 resolution provides a substantial boost in performance for the other sign class too (see Figure 12). Both Precision and Recall scores improve significantly, with the highest Precision score reaching 91.6% (YOLOv11m) and the highest Recall score reaching 58.4% (YOLOv8l). This indicates that the higher resolution provides the models with the detail needed to better handle the visual variations of this class, leading to more consistent and accurate detection. The improvement is a testament to the fact that providing more visual context and detail helps the models generalize better to a wider range of object appearances.

In contrast to the previous classes, the car class, being a relatively large and common object, shows solid performance at the 576 × 640 resolution. Precision scores are generally high, ranging from 60.0% to 85.1%, while Recall scores are in the 50–60% range. This suggests that the models are accurate when they make a detection, but a significant portion of cars are still being missed. The YOLOv8s and YOLOv11l models show the best trade-off at this resolution, with an F1-score of 71.6% and 71.5%, respectively.

**Figure 13:**
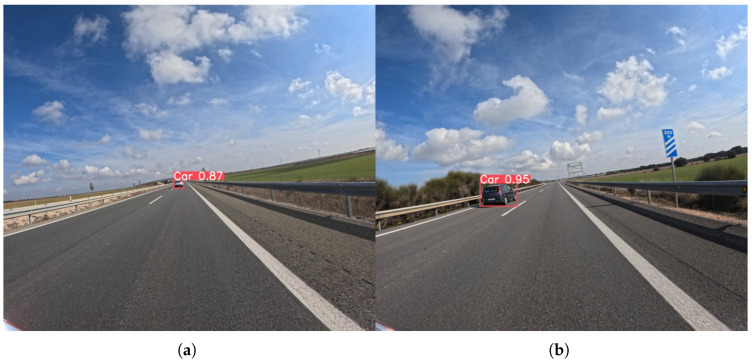
Representative outputs of the YOLOv8l model with an input size of 1120 × 1280, which demonstrates the best overall performance in the car detection task. An example (**a**) demonstrating successful detection of a distant car, and (**b**) showing accurate detection of a closer car on the highway.

As demonstrated in Figure 13, at the higher resolution, the performance for the car class improves across the board. The Precision scores increase to the high 80s, with YOLOv12s reaching a peak of 89.9%, and the Recall scores also see a boost, with YOLOv8l achieving the best Recall at 83.4%. This confirms that even for large, common objects, higher resolution provides additional features that lead to more robust and reliable detection. The improved performance means that models can now both accurately identify a greater number of cars and do so with fewer false positives.

The truck class, similarly to cars, performs well at the lower resolution. Precision scores are consistently high, ranging from 73.3% to 91.3%, while Recall scores are also strong, ranging from 76.3% to 90.0%. This indicates that trucks are generally easy for the models to detect and identify, likely due to their large size and distinct shape. The YOLOv11l model achieves the highest Precision (91.3%), while the YOLOv8l model achieves the highest Recall (90.0%), showcasing a good balance between the two metrics.

**Figure 14:**
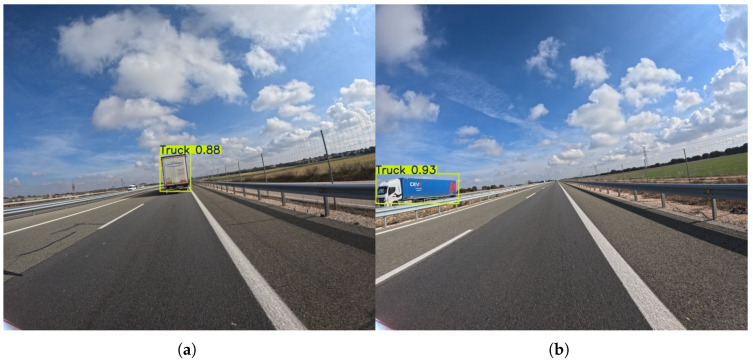
Representative outputs of the YOLOv8s model with an input size of 1120 × 1280, which demonstrates the best overall performance in the truck detection task. An example (**a**) demonstrating successful detection of a truck in an adjacent lane, shown at a moderate distance, and (**b**) showing accurate detection of a distant truck.

With the resolution increase to 1120 × 1280, the models maintain their strong performance (see Figure 14). Precision and Recall scores remain high, with some models even exceeding 90% in both metrics. Similarly to the lower resolution, the YOLOv11l model achieves the highest Precision (92.4%), and the YOLOv8l model achieves the highest Recall (95.5%). This confirms that for large and well-defined objects, the models’ performance is already at a high level at a lower resolution. However, the higher resolution provides a small but notable boost, making the identifications even more reliable and accurate.

Similarly, the guardrail class demonstrates exceptional performance at the 576 × 640 resolution, with both Precision and Recall scores consistently in the high 90s across all models. It is underlined that the lowest Precision score is 93.5%, and the lowest Recall score is 96.1%, both demonstrated by the YOLOv12s model. These near-perfect scores indicate that models are highly effective at detecting and localizing guardrails, which are large, continuous, and visually distinct objects. Since the F1-scores range from 94.8% (YOLOv12s) to 96.5% (YOLOv12m), there is virtually no trade-off between Precision and Recall for this class, as the models can both find nearly all instances and make very few false positive detections.

**Figure 15:**
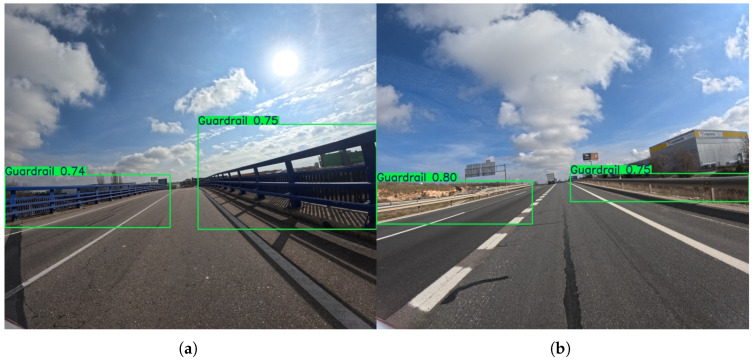
Representative outputs of the YOLOv11l model with an input size of 1120 × 1280, which demonstrates the best overall performance in the guardrail detection task. An example (**a**) demonstrating the challenge of sun glare, and (**b**) showing detection of guardrails in a clear scene.

As demonstrated in Figure 15, at the higher resolution, the performance for guardrails remains outstanding. The scores stay in the high 90s, with the best Precision reaching 97.9% (YOLOv11s) and the best Recall reaching 96.7% (YOLOv8s). F1-scores remain high, with all models exceeding 95% and YOLOv12m reaching a peak of 97.3%. The marginal gains confirm that for objects that are already easily identifiable, the benefit of increased resolution is minimal. The models are already operating at a near-optimal level, demonstrating that a higher resolution is not always necessary for classes with such distinct visual characteristics.

Lastly, similarly to guardrails, the road class exhibits near-perfect performance at the lower 576 × 640 resolution, with all models achieving both Precision and Recall scores of over 98%. This highlights that the models are extremely proficient at identifying the road surface, which is a large, consistent, and easily distinguishable feature in the images. The consistently high scores across all models, regardless of size, indicate that even the most lightweight architectures are sufficient for detecting this class.

**Figure 16:**
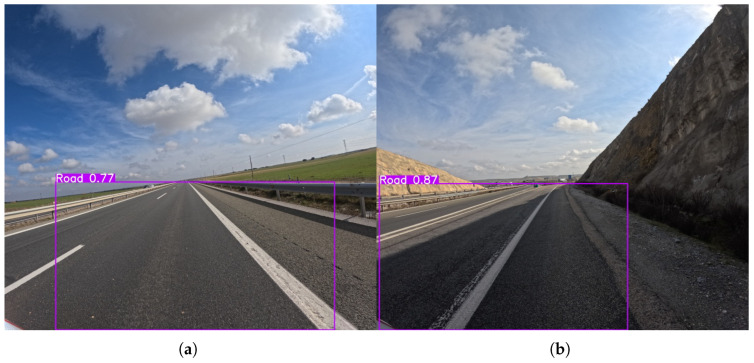
Representative outputs of the YOLOv11s model with an input size of 1120 × 1280, which demonstrates the best overall performance in the road detection task. Output examples demonstrating road detection on a (**a**) multi-lane highway and (**b**) single-lane road.

The performance for the road class at the 1120 × 1280 resolution remains similarly high as presented in Figure 16, with both Precision and Recall scores staying in the high 90s. However, while the overall performance remains exceptionally high, the gains from increasing resolution are negligible. For instance, the best F1-score for the road class at the lower resolution was 99.2% (YOLOv8l), which only increased by 0.1% to 99.3% (YOLOv12s) at the higher resolution. These results reinforce the finding from the lower resolution analysis: for easily identifiable, large, and consistent classes, the models’ performance is already at its peak, and increasing the resolution provides negligible additional benefit.

#### 4.3.3. Inference Time Analysis

**Figure 17:**
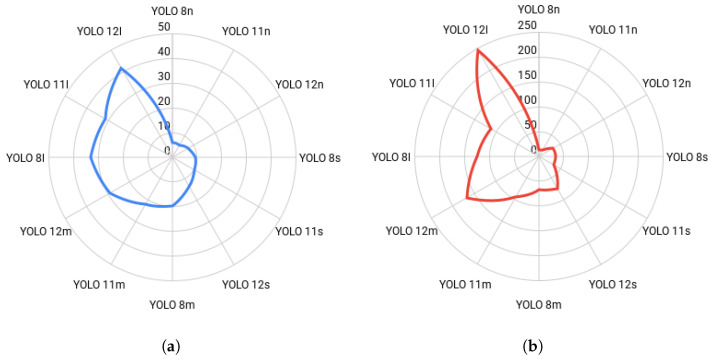
Inference time comparison (milliseconds) by model size and resolution. The charts illustrate the latency trade-off: (**a**) 576×640 resolution, showing low latency for nano models. (**b**) 1120×1280 resolution, demonstrating the sharp, non-linear increase in processing time at the higher input size, especially for the large YOLO architectures.

The final consideration in evaluating the YOLO detectors is the inference time, which is crucial for real-time applications such as autonomous driving or infrastructure monitoring. Inference time (measured in milliseconds) directly relates to the computational cost and speed of the corresponding model. Figure 17 presents the inference times for all networks across both input resolutions.

A clear and expected correlation exists between model size, input resolution, and inference time: larger architectures and higher resolutions result in significantly slower inference speeds. At the lower 576 × 640 resolution, the nano detectors are exceptionally fast, with YOLOv11n being the quickest at 5.7 ms (approximately 175 frames per second). The speed progressively drops as the system size increases, reaching 41.3 ms for the largest YOLOv12l model. This initial trend, visually summarized in Figure 17a, highlights the strong advantage of the nano and small architectures for speed-critical, real-time applications where detection latency must be minimal.

The transition to the 1120 × 1280 resolution results in a dramatic, non-linear increase in inference time across all frameworks, as illustrated by the sharp jump in values shown in Figure 17b. This increase is most pronounced in the large networks, where YOLOv12l’s time skyrockets to 245.2 ms (nearly a quarter of a second), a 494% increase from its 576 × 640 time. While the nano models remain the fastest at this resolution (YOLOv8n at 13.8 ms), their speed advantage relative to the smaller input size is severely curtailed. This analysis demonstrates a critical trade-off: the substantial accuracy gains achieved with higher resolution, particularly for small objects, come at a severe cost in terms of computational speed, pushing many large models outside the requirements for hard real-time performance.

When considering the optimal balance between accuracy and speed, architectures in the medium to large categories, such as YOLOv8l (32.9 ms, mAP50 = 60.7%) and YOLOv8m (19.6 ms, mAP50 = 59.9%), offer a compelling point of compromise at the 576 × 640 resolution. However, when the higher accuracy offered by the 1120 × 1280 resolution is critical (e.g., detecting danger signs with mAP50 > 90%), a faster medium model like YOLOv8m (67.2 ms) must be selected, accepting a significantly higher latency to meet the required precision standards. Conversely, if pure speed is the main priority, the YOLOv11n network consistently offers near-minimal latency across both input resolutions, thus making it the most resource-efficient choice.

## 5. Discussion

The experimental evaluation provided in this work highlights several important findings regarding the proposed dataset and the performance of state-of-the-art object detection systems. Overall, the results confirm that model capacity and input resolution are critical factors for successfully detecting infrastructure-related objects. Larger YOLO variants (e.g., medium and large models) consistently outperformed their lighter counterparts, which is in line with observations from other computer vision benchmarks.

One notable finding is the large performance gap between easily detectable classes (e.g., guardrail and road) and more challenging small-scale objects (e.g., signs, bollards, and delineators). This discrepancy underlines the inherent difficulty of detecting small, visually diverse, and sometimes occluded objects within driving scenes. Increasing the input resolution significantly improved performance for some classes, with gains over 40% in mAP scores, showing that small objects need a higher pixel density for reliable detection.

The results also demonstrate that DORIE offers realistic challenges similarly to those encountered in real-world infrastructure monitoring. Unlike datasets that focus primarily on vehicles and pedestrians, our collection emphasizes safety-critical static infrastructure, which introduces new detection difficulties not adequately represented elsewhere. These challenges make the dataset valuable for advancing research in specialized domains, such as automated road safety assessment and intelligent maintenance systems.

While the DORIE dataset and the conducted experiments provide a solid foundation for real-time infrastructure monitoring research, several limitations should be acknowledged to ensure a balanced interpretation of the results. First, the current benchmark is restricted to the YOLO family of detectors (versions 8, 11, and 12). Future work will expand the evaluation to include vision transformers (ViTs), DETR-like architectures, and temporal fusion networks to assess the generalizability of the dataset across paradigms. Second, the dataset size and geographic scope remain limited to a single motorway segment and a single forward-facing RGB camera configuration. While this ensures controlled data quality, it constrains diversity in lighting, road topology, and sensor viewpoints. Expanding DORIE to additional routes, weather conditions, and sensor modalities (e.g., stereo, thermal, or depth cameras) will enhance its representativeness and applicability. Future improvements could also be achieved by exploring techniques for small object detection, such as multi-scale feature aggregation, super-resolution pre-processing, and transformer-based models that better capture long-range dependencies. Additionally, our dataset could be extended with targeted data collection and synthetic augmentation, nighttime recordings, adverse weather conditions, and video-level annotations to enable temporal reasoning and robustness analysis in more diverse scenarios.

## 6. Conclusions

This work presented DORIE—the Dataset of Road Infrastructure Elements—along with a deployment-oriented benchmark designed for patrol-vehicle monitoring and inventory maintenance. The dataset targets static, safety-relevant roadside assets and traffic signs that are underrepresented in existing benchmarks. We aligned the data design with realistic operational viewpoints, defined an evaluation protocol that emphasizes small-object performance and real-time constraints, and systematically benchmarked recent YOLO families across model sizes and input resolutions, reporting both detection quality and per-frame latency to expose accuracy–throughput trade-offs. Practical implementation assets are also provided to facilitate training, evaluation, and integration into inventory pipelines.

Future work will expand the dataset’s breadth and operational value. We plan to broaden geography and road types, include nighttime and adverse weather, add asset classes such as terminals, crash cushions, and work-zone furniture, and provide video-level annotations to enable temporal consistency studies. We will pursue geospatial grounding by fusing detections with GPS/IMU and visual odometry to produce map-accurate asset locations with uncertainty estimates and change logs, allowing evaluation of geolocation error alongside detection metrics. On the modeling side, we will investigate small-object-oriented designs (for example, additional low-level heads and tiled inference), class-imbalance strategies, stronger augmentation, and semi- or active-learning loops supplemented by synthetic data for rare categories; metric reporting will be broadened to mAP50:95 with size-stratified precision–recall analyses. For deployment, we will profile accuracy, latency, and power on representative edge hardware and runtimes (e.g., Jetson, CPU-only, TensorRT/ONNX), quantify pre/post-processing and memory footprints, and develop human-in-the-loop review tools and quality-assurance dashboards that convert detections into auditable inventory updates.

Taken together, DORIE, the evaluation protocol, and the baselines provide a foundation for reliable, scalable, and timely inventorying of road infrastructure. We expect these resources to catalyze research on small-object detection under operational constraints and to accelerate real-world adoption across transportation agencies and, more broadly, other infrastructure domains where robust asset registries are essential.

## Figures and Tables

**Table 1:** Number of annotated instances per class for each dataset split.

Class	Train Set	Validation Set	Test Set	Total Instances
Bollard	303	81	93	**477**
Delineator	1227	291	378	**1896**
Prohibitory sign	152	32	41	**225**
Danger sign	75	14	16	**105**
Mandatory sign	82	22	27	**131**
Other sign	125	28	40	**193**
Car	436	117	140	**693**
Truck	358	89	110	**557**
Guardrail	1048	257	330	**1635**
Road	602	150	188	**940**
**Total instances**	**4408**	**1081**	**1363**	**6852**

**Table 2:** Model complexity metrics: A comparison of parameter counts (par) in millions and computational cost (flops) in billions for the YOLOv8, YOLOv11, and YOLOv12 architectures at the base 576 × 640 input resolution.

	8n	11n	12n	8s	11s	12s	8m	11m	12m	8l	11l	12l
**par**	3.2	2.6	2.6	11.2	9.4	9.3	25.9	20.1	20.2	43.7	25.3	26.4
**flops**	8.7	6.5	6.5	28.6	21.5	21.4	78.9	68.0	67.5	165.2	86.9	88.9

**Table 3:** Performance comparison of the various YOLO detection models with an input size of 576 × 640, evaluated by their mAP50 scores.

	8n	11n	12n	8s	11s	12s	8m	11m	12m	8l	11l	12l
**All**	48.2	47.1	46.8	56.2	56.9	54.9	59.9	58.3	57.3	**60.7**	60.0	58.3
**Bol**	24.1	21.7	25.1	31.9	37.9	31.1	37.6	**40.8**	36.1	39.6	38.4	39.1
**Del**	21.3	22.7	23.3	29.6	30.9	30.0	35.5	34.2	33.5	33.9	**36.1**	33.7
**Pro**	36.9	31.4	32.0	48.6	48.9	44.9	46.1	**52.8**	47.6	48.7	52.0	47.8
**Dan**	6.38	4.49	6.93	14.2	16.4	13.8	24.2	16.3	14.1	**31.0**	21.5	22.8
**Man**	40.3	35.3	28.2	50.1	47.0	44.9	53.5	53.0	49.2	**53.7**	52.6	49.7
**Oth**	17.5	15.1	15.3	31.1	29.5	30.8	**39.2**	28.7	34.4	38.4	39.2	36.2
**Car**	58.4	58.7	54.7	69.5	69.5	66.9	**73.8**	68.4	70.1	71.9	72.2	67.3
**Trk**	81.5	85.9	86.9	91.1	91.5	90.0	92.8	92.7	91.6	**93.6**	92.1	90.7
**Grd**	97.3	97.1	97.0	97.2	**97.6**	97.3	97.1	97.1	97.2	97.4	96.9	97.2
**Rd**	98.3	98.4	98.4	98.9	**99.3**	99.0	98.8	98.9	99.1	98.9	98.9	98.9

**Table 4:** Performance comparison of the various YOLO object detection models with an input size of 1120 × 1280, evaluated by their mAP50 scores.

	8n	11n	12n	8s	11s	12s	8m	11m	12m	8l	11l	12l
**All**	72.5	70.5	71.0	80.0	82.1	83.1	84.0	84.7	84.2	**86.2**	85.1	82.1
**Bol**	61.8	62.6	59.3	74.4	73.1	74.6	75.8	73.1	73.4	71.1	**76.7**	69.0
**Del**	57.5	57.7	56.2	66.2	68.1	69.1	67.8	74.2	73.0	75.8	**76.0**	70.2
**Pro**	57.7	57.4	57.5	69.3	72.0	75.7	77.6	77.1	75.5	**83.1**	81.0	74.1
**Dan**	59.1	45.3	63.6	71.8	85.5	90.4	92.1	85.7	91.1	**92.7**	87.7	85.3
**Man**	71.1	60.8	60.3	80.0	80.3	83.6	86.2	**86.2**	83.2	85.6	82.8	84.3
**Oth**	47.2	47.9	41.7	59.3	62.3	60.6	63.5	68.5	67.0	**71.5**	62.5	61.1
**Car**	82.1	83.2	83.3	86.3	88.2	87.6	88.5	89.8	89.1	**91.4**	91.3	89.3
**Trk**	92.3	95.0	92.7	**96.8**	94.4	92.7	92.2	96.0	93.8	94.8	96.1	93.1
**Grd**	97.5	97.1	97.5	97.6	97.4	97.5	97.2	97.3	97.4	97.3	**97.7**	96.8
**Rd**	98.6	98.4	98.2	98.7	**99.4**	99.0	98.9	99.2	98.9	99.1	99.2	97.2

**Table 5:** Performance comparison of the various YOLO detection models with an input size of 576 × 640, evaluated by their mAP50:95 scores.

	8n	11n	12n	8s	11s	12s	8m	11m	12m	8l	11l	12l
**All**	33.4	32.6	32.1	37.0	38.3	36.7	**40.0**	38.5	38.2	39.6	39.3	38.5
**Bol**	13.7	13.4	13.7	17.2	19.0	15.8	**20.0**	19.7	19.4	19.3	19.2	19.8
**Del**	9.4	9.2	9.7	13.0	13.1	12.7	**16.3**	15.5	14.9	15.3	15.6	14.7
**Pro**	20.6	15.9	17.3	24.4	25.2	24.2	27.0	28.0	24.3	**28.5**	26.7	25.8
**Dan**	2.5	2.0	2.0	5.7	5.7	6.4	7.9	7.4	6.8	**14.3**	8.8	8.6
**Man**	26.1	21.8	17.2	28.5	30.9	26.8	**34.8**	32.5	33.1	30.3	30.2	28.1
**Oth**	7.6	6.7	6.7	13.6	13.8	14.2	17.4	11.6	13.2	14.9	17.3	**18.0**
**Car**	30.9	32.9	30.9	38.0	40.5	38.6	**42.3**	40.2	38.6	40.4	42.0	39.1
**Trk**	52.3	52.7	53.1	56.7	60.3	56.3	**61.1**	58.3	58.6	60.3	59.1	58.6
**Grd**	75.7	75.2	75.1	76.2	**77.1**	75.4	75.9	74.4	75.5	75.4	77.1	75.3
**Rd**	95.7	95.8	95.5	96.9	**97.6**	97.0	97.3	97.0	97.5	97.1	97.0	97.0

**Table 6:** Performance comparison of the various YOLO object detection models with an input size of 1120 × 1280, evaluated by their mAP50:95 scores.

	8n	11n	12n	8s	11s	12s	8m	11m	12m	8l	11l	12l
**All**	40.5	40.9	41.0	46.1	48.9	47.6	48.3	**50.3**	48.8	**50.3**	50.1	48.3
**Bol**	30.2	30.6	29.5	37.8	39.3	38.4	38.5	39.7	37.8	39.2	**41.3**	38.1
**Del**	26.0	25.3	24.7	30.0	31.4	30.2	31.0	33.4	32.2	**34.0**	33.7	31.9
**Pro**	30.4	30.8	32.8	39.4	42.2	42.5	41.0	43.2	42.5	**45.0**	41.2	41.2
**Dan**	22.1	20.5	26.6	36.4	47.0	37.8	48.3	52.5	49.0	**55.2**	51.2	49.6
**Man**	36.7	37.1	37.4	39.2	48.5	47.1	50.6	48.6	47.9	51.0	**52.1**	51.9
**Oth**	21.5	24.0	19.6	27.4	28.4	28.0	28.8	**32.5**	30.2	30.0	29.3	28.4
**Car**	46.7	48.9	47.7	53.4	**55.6**	55.0	51.6	54.7	53.5	54.8	55.1	53.8
**Trk**	62.7	63.7	62.3	**65.8**	63.4	64.8	62.9	65.5	63.8	63.2	65.7	62.9
**Grd**	74.8	74.6	75.9	76.3	**77.6**	77.0	75.9	77.2	76.6	75.2	75.9	74.1
**Rd**	95.5	95.5	95.3	97.4	97.7	96.9	96.5	97.6	96.7	97.7	**98.1**	93.0

**Table 7:** Performance comparison of the various YOLO detection models with an input size of 576 × 640, evaluated by their F1-scores.

	8n	11n	12n	8s	11s	12s	8m	11m	12m	8l	11l	12l
**All**	54.6	54.9	53.4	62.5	63.6	60.3	64.8	61.1	53.4	66.2	**67.0**	63.5
**Bol**	30.7	30.3	30.2	37.9	45.8	36.2	45.2	**50.7**	31.8	43.2	45.6	47.0
**Del**	29.1	29.1	31.9	34.9	39.6	38.2	**43.1**	41.8	26.6	40.2	40.8	39.9
**Pro**	41.7	41.3	36.0	55.6	**59.9**	51.6	50.0	53.1	47.3	54.7	56.6	55.0
**Dan**	9.8	10.8	10.6	11.2	18.4	25.0	**40.8**	8.7	8.3	39.8	29.1	25.0
**Man**	51.7	41.6	35.4	54.3	57.3	49.9	57.9	58.1	49.9	**61.0**	60.6	51.8
**Oth**	14.3	13.1	17.1	26.0	32.6	30.6	42.5	31.6	25.3	43.3	**47.0**	35.5
**Car**	60.9	62.3	60.4	**71.6**	71.3	67.6	70.9	68.2	60.3	69.8	71.5	69.5
**Trk**	77.0	82.0	77.3	86.8	84.3	81.3	85.3	86.2	86.5	87.3	**88.8**	87.4
**Grd**	96.3	95.1	95.0	**96.5**	96.0	94.8	95.6	96.9	**96.5**	96.4	96.1	95.1
**Rd**	98.7	98.8	98.8	98.8	99.0	98.9	98.9	98.7	99.0	**99.1**	98.8	98.9

**Table 8:** Performance comparison of the various YOLO detection models with an input size of 1120 × 1280, evaluated by their F1-scores.

	8n	11n	12n	8s	11s	12s	8m	11m	12m	8l	11l	12l
**All**	74.7	71.1	72.3	80.4	80.9	82.7	82.7	**83.7**	82.4	83.5	83.5	79.3
**Bol**	66.9	65.0	60.2	76.1	78.3	78.5	79.5	74.1	76.1	75.2	**80.7**	73.7
**Del**	60.0	59.9	60.8	64.6	66.4	66.4	64.1	68.9	67.6	**69.6**	**69.6**	68.6
**Pro**	57.3	57.2	60.1	71.3	71.5	74.2	79.6	77.9	75.4	81.4	**81.8**	73.5
**Dan**	70.6	39.4	66.6	74.3	70.5	86.1	82.0	84.1	**86.7**	83.3	77.9	78.6
**Man**	71.9	61.1	55.7	78.9	81.6	85.7	86.1	**87.8**	82.6	83.8	84.0	82.0
**Oth**	46.7	46.5	47.5	58.9	66.1	59.0	69.1	68.2	64.6	**70.4**	65.6	59.8
**Car**	77.7	78.2	78.9	81.7	**84.7**	82.6	81.4	84.1	82.7	82.3	82.8	81.4
**Trk**	63.6	89.4	86.2	**91.2**	86.4	89.5	82.4	88.5	85.7	88.2	90.6	88.9
**Grd**	96.5	96.7	96.6	97.1	**97.3**	97.1	96.7	96.3	97.3	95.0	96.7	96.4
**Rd**	98.9	98.9	**99.2**	97.6	98.0	**99.2**	95.6	98.8	98.8	98.4	98.6	98.2

## Data Availability

The dataset of this work is available online at the following link: https://doi.org/10.5281/zenodo.17277466 (accessed date 6 October 2025).
